# Supplementary material for: COZOID: contact zone identifier for visual analysis of protein-protein interactions
Source: BMC Bioinformatics. 2018 Apr 6;19:125. doi: 10.1186/s12859-018-2113-6 (PMC5889581; doi:10.1186/s12859-018-2113-6)
Supplement: Supplementary file 2 — Software build. Executable binary file of the software tool. (ZIP 118,784 kb) [file 12859_2018_2113_MOESM2_ESM.zip › cozoid/java/docs/beansbinding-1.2.1-doc.zip!/org/jdesktop/beansbinding/Validator.Result.html]

Validator.Result (Beans Binding)


---


|  |  |  |
| --- | --- | --- |
| **Overview** \| **Package** \| **Class** \| **Use** \| **Tree** \| **Deprecated** \| **Index** \| **Help** | |  |
| **PREV CLASS**   NEXT CLASS | **FRAMES**    **NO FRAMES**     **All Classes** |  |
| SUMMARY: NESTED \| FIELD \| CONSTR \| METHOD | DETAIL: FIELD \| CONSTR \| METHOD |  |


---


## org.jdesktop.beansbinding Class Validator.Result

```
java.lang.Object
  org.jdesktop.beansbinding.Validator.Result
```

**Enclosing class:**: Validator<T>

---

``` public class Validator.Result extends java.lang.Object ```

An instance of `Result` is returned from a `Validator's`
`validate` method to indicate an invalid value.

A `Result` can contain an error code and/or description.
These values are for your own reporting purposes and are not used
internally.

---

**Constructor Summary**

`Validator.Result(java.lang.Object errorCode,
java.lang.String description)`
  
          Creates a `Result` with the given error code and description.


| **Method Summary** | |
| --- | --- |
| `java.lang.String` | `getDescription()`             Returns a description of the validation result, which may be `null`. |
| `java.lang.Object` | `getErrorCode()`             Returns the error code for the result, which may be `null`. |
| `java.lang.String` | `toString()`             Returns a string representation of the `Result`. |

**Methods inherited from class java.lang.Object**

`clone, equals, finalize, getClass, hashCode, notify, notifyAll, wait, wait, wait`

**Constructor Detail**

### Validator.Result

```
public Validator.Result(java.lang.Object errorCode,
                        java.lang.String description)
```

:   Creates a `Result` with the given error code and description.

    **Parameters:**: `errorCode` - an error code for this `Result`, may be `null`: `description` - a textual description of the `Result`, may be `null`


**Method Detail**

### getErrorCode

```
public java.lang.Object getErrorCode()
```

:   Returns the error code for the result, which may be `null`.

    :   **Returns:**: the error code

---


### getDescription

```
public java.lang.String getDescription()
```

:   Returns a description of the validation result, which may be `null`.

    :   **Returns:**: the description

---


### toString

```
public java.lang.String toString()
```

:   Returns a string representation of the `Result`. This
    method is intended to be used for debugging purposes only, and
    the content and format of the returned string may vary between
    implementations. The returned string may be empty but may not
    be `null`.

    :   **Overrides:**: `toString` in class `java.lang.Object`
    :   **Returns:**: a string representation of this `Result`


---


|  |  |  |
| --- | --- | --- |
| **Overview** \| **Package** \| **Class** \| **Use** \| **Tree** \| **Deprecated** \| **Index** \| **Help** | |  |
| **PREV CLASS**   NEXT CLASS | **FRAMES**    **NO FRAMES**     **All Classes** |  |
| SUMMARY: NESTED \| FIELD \| CONSTR \| METHOD | DETAIL: FIELD \| CONSTR \| METHOD |  |


---
